# A model to promote the mental health of radiation therapy students throughout their training

**DOI:** 10.4102/hsag.v30i1.2969

**Published:** 2025-09-30

**Authors:** Louisa Mokoena, Karien Henrico, Emmerentia du Plessis

**Affiliations:** 1 Department of Medical Imaging and Radiation Sciences, Faculty of Health Sciences, University of Johannesburg, Johannesburg, South Africa; 2 Department of Emergency Medical Care, Faculty of Health Sciences, University of Johannesburg, Johannesburg, South Africa; 3 Department of Nursing, Faculty of Health Sciences, North-West University, Potchefstroom, South Africa

**Keywords:** supportive learning environment, mental health, radiation therapy lecturers, clinical tutors, students

## Abstract

**Background:**

Clinical placement constitutes an essential aspect of the radiation therapy (RT) undergraduate programme, but students have expressed that they often experience challenges during their training. There is limited guidance for RT lecturers and clinical tutors on how to support these students. Developing a model to promote their mental health during training was therefore essential.

**Aim:**

This article outlines the development and evaluation process of a model aimed at promoting mental health support for RT students during their academic training programme.

**Setting:**

This model is intended for RT lecturers and clinical tutors involved in teaching RT students throughout their training.

**Methods:**

A qualitative, exploratory, descriptive, contextual and theory-generating study design was employed for the development of the model. The process of model development comprised four key steps: concept analysis, the formulation of relationship statements, model description and evaluation. The evaluation criteria used to assess the model included clarity, simplicity, generality, accessibility and significance.

**Results:**

The central concept identified was: ‘facilitation of a supportive learning environment’. The concept was defined, classified and placed into relationship statements leading to the development, description and evaluation of the model.

**Conclusion:**

The model can be used by RT lecturers and clinical tutors to promote mental health support for RT students during their academic training.

**Contribution:**

The contribution of the model is to strengthen the RT students’ mental health and empower them to develop their clinical skills in a supportive and nurturing learning environment.

## Introduction

In South Africa, students enrolled in radiation therapy (RT) programmes engage in academic instruction at universities alongside practical training within radiation oncology departments. Clinical placements are essential for developing practical skills, as they provide valuable hands-on experience in real-world healthcare settings (Kumsa et al. 2022; Lundvall, Dahlström & Dahlgren 2021). During these placements, students enhance their understanding through direct interactions with patients under the guidance of clinical tutors, effectively bridging the gap between theoretical knowledge and practical application (Smith et al. 2014; Hazell, Lawrence & Friedrich-Nel 2020).

A review of 23 quantitative studies revealed that clinical placements, while essential for practical learning, expose healthcare students to a myriad of stressors which significantly heighten their vulnerability to psychological distress (Pulido-Martos et al. 2012). Several factors contribute to the challenges faced by students in demanding clinical settings, which are often characterised by high-pressure situations. The need to balance rigorous academic requirements with patient care responsibilities can be particularly difficult for many students (Jeyandrabalan et al. 2022). Furthermore, McPake (2021) reported that negative attitudes or behaviours from radiographers could significantly hinder students’ learning during placements. Such experiences may lead to feelings of dread, anxiety and uncertainty. Many students expressed that being ‘brushed off’ made them feel ‘unwanted’. Additionally, McPake (2021) found that even a single unpleasant experience could severely undermine a student’s confidence and negatively impact future placements.

### Problem statement

The field of cancer care is widely recognised as both stressful and complex, with challenges that can be overwhelming for the individuals involved (Cohen et al. 2010). For RT students, this demanding environment can create significant levels of pressure because they are required to navigate a landscape filled with high-stakes situations that feel both novel and foreign to them, which can be quite daunting (Hughes & Byrom 2019; Probst et al. 2014). In light of these challenges, Chamunyonga et al. (2020) strongly advocate for the importance of equipping students with practical skills to effectively handle stress. This support is vital not only for managing the pressures they face in clinical settings but also for coping with stressors that can arise in everyday life. Furthermore, Khine, Harrison and Flinton (2024) expressed that the quality of the student’s clinical experience plays a critical role in shaping their learning outcomes and can deeply influence their decision to continue or leave their programme. When students receive effective guidance and support during clinical practice, they are more likely to feel confident and engaged, enhancing their overall educational journey. In contrast, poor clinical experience can lead to frustration and feelings of inadequacy, increasing the risk of attrition.

The researcher reviewed the existing literature to identify methods for supporting students during their training. However, most studies focused on international contexts. For example, strategies for supporting medical radiation sciences students in Australia were outlined by Chamunyonga et al. (2020), while Cohen and Legg (2019) examined non-academic sources of stress among radiologic science students in the United States of America. There was, however, limited literature specifically addressing the promotion of mental health among South African RT students during their training. To fill this gap, a model was developed based on the findings of the study, aimed at guiding RT lecturers and clinical tutors in supporting students’ mental health throughout their clinical education.

### Research purpose

This study aims to detail the development, description, and evaluation of a model designed as a framework for promoting mental health support among RT students throughout their academic training programme.

### Research objectives

- To explain how the central concepts used in the model development were derived.
- To describe the relationship between these concepts.
- To outline the development of a model that serves as a framework for promoting mental health support among RT students throughout their academic training.
- To detail how the model was evaluated by the evaluators.

## Definition of key concepts

The key concepts of this study were defined as follows:

### Model

A symbolic representation of empirical experiences, utilising words, diagrams or graphics, when expressed in written form, models constitute a type of knowledge derived from these empirical patterns (Chinn & Kramer 2018). A model is a systematic description of interrelated concepts to provide guidance on a specific problem in a specific context, and it is testable and can be modified through processes of verification (Chinn & Kramer 2018). In this study, a model is a frame of reference aimed at guiding RT lecturers and clinical tutors in promoting mental health support among RT students. This model encompasses various strategies that can help create a supportive learning environment for students in the RT field.

### Facilitation

A dynamic, interactive process that helps promote health by creating a positive environment and mobilising resources, and identifying and bridging obstacles to health promotion (University of Johannesburg 2012). Facilitation in this study refers to the role that RT lecturers and clinical tutors must play to promote mental health support of RT students during their academic training.

### Radiation therapy student

An individual who has enrolled in a formal educational programme in radiation therapy, structured to facilitate the attainment of specific competencies or qualifications required for professional practice in the field.

### Radiation therapist

A professional who oversees the daily administration of safe and accurate radiation doses to cancer patients. The individual’s responsibilities include preparing and planning radiation therapy treatments, as well as providing both clinical and psychosocial support to patients throughout their treatment journey. Additionally, this role involves conducting immediate post-treatment evaluations, monitoring for side effects and facilitating appropriate referrals to other healthcare professionals to ensure that all patient needs are met (Coffey, Naseer & Leech 2022; Washington 2016).

### Radiation therapy clinical tutor

A radiation therapy clinical staff member who is knowledgeable in the actual radiation therapy treatment planning and delivery equipment and techniques. This individual plays an active role in the clinical education of the RT student, ensuring they are competent to practise and are therefore responsible for the student’s progress in the clinical setting (International Atomic Energy Agency 2014). Experienced clinical radiation therapists are expected to support the students in gaining an understanding of radiation therapy principles and in developing their technical and psychosocial skills.

### Mental health

The Latin word men and the Greek word nous, are the roots of the term mental health (Braithwaite 2006), which is defined as the state in which an individual can maintain meaningful relationships, perform socially and culturally expected roles, adapt to change, regulate emotions, and reflect positively on their thoughts and actions, resulting in a sense of self-worth, control, and understanding of themselves and their environment (Bhugra, Till & Sartorius, 2013). Additionally, mental health involves an internal self-care process that emphasises self-awareness and self-regulation. It aims to help the person achieve balance in their feelings, thoughts and behaviours, both within themselves and in their interactions with others. This process seeks to attain an ideal state of well-being and the absence of mental disorders in accordance with universal values and symptoms, as well as biological, social, psychological and environmental factors (Coronel-Santos & Rodríguez-Macías 2022). In this study, mental health is viewed as a dynamic process that relates to: (1) how RT students think, (2) how they react to everyday situations and (3) how they interact with others. Additionally, mental health pertains to RT students’: (1) ability to recognise their strengths, (2) capacity to function effectively and make a positive contribution in their departments and to their patients’ lives and (3) ability to cope with the normal stresses and challenges of working in a radiation oncology department.

## Research methods and design

### Research design

A qualitative, contextual, exploratory, descriptive and theory-generating research design was used to develop the model (Busetto, Wick & Gumbinger 2020; Chinn & Kramer 2015; Creswell 2023; Gray & Grove 2021; Moser & Korstjens 2017; Rendle et al. 2019; Walker & Avant 2014).

### Method

The theory development steps outlined by Chinn and Kramer (2018) were used. The first step involved identifying the concept, followed by classifying the concept according to the framework provided by Dickoff, James and Wiedenbach (1968). In the second step, the relationships between the concepts were described. The third step entailed the model description, while the fourth step focused on the model evaluation (Chinn & Kramer 2018).

### Measures to ensure trustworthiness

Lincoln and Guba’s (1985) techniques, including credibility, transferability, dependability and confirmability, were followed throughout the data collection and analysis procedures to ensure the trustworthiness of this research study. Credibility was achieved by prolonged engagement, persistent observation, triangulation and member-checking (Amankwaa 2016). Transferability was ensured by providing a detailed description of the research study’s setting, demographic information of the participants and verbatim quotes from the participants. This information was provided so that it can be easily applied in other studies (Amin et al. 2020). Dependability was achieved by providing details of the context of this study, together with an audit trail of the data collection and analysis procedures to enable other researchers to repeat the study so that they can evaluate the accuracy and whether or not the findings, interpretations and conclusions are supported by the data (Lincoln & Guba 1985). Lastly, confirmability was achieved by providing a detailed account of how interpretations and conclusions of the research findings were established (Holloway & Wheeler 2010).

### Ethical considerations

The data collection for this study began after obtaining approval from University of Johannesburg’s Higher Degrees Committee (HDC-01-138- 2023) as well as the Research Ethics Committee (clearance no: REC-1031-2021). Permission was also sought from the Higher Degrees Committees of the Faculty of Health Sciences at the two universities that offer education in radiation therapy, i.e., CPUT/HWS-REC 2022/S8. Furthermore, the researcher sought permission from the heads of departments at: (1) the three universities offering radiation therapy programmes and (2) the departments where the clinical tutors were employed. In addition to this, the four ethical principles (autonomy, non-maleficence, justice and beneficence), which researchers are required to adhere to when conducting research studies (Dhai & McQuoid-Mason 2010), were followed to uphold the integrity and ethical standards of the research study. Autonomy was achieved by ensuring that all participants had all the information they needed to make an informed decision concerning participation in this study. Privacy was achieved by ensuring that the discussion sessions with the participants were held at times that were suitable to all the participants. Additionally, at the beginning of each discussion, the participants were informed that they could share or withhold any information they were not comfortable sharing during the discussions. Anonymity in the study was meticulously maintained by implementing measures that prevented the researcher and any individuals reviewing the research findings from associating specific responses with individual participants. This careful approach ensured that personal identities remained completely concealed, safeguarding the privacy of all involved (Babbie 2021). To maintain the confidentiality of participants, all collected data, including backups, was securely stored on password-protected electronic devices. Access to this data was limited to the researcher and the supervisors. Additionally, participants were assured that their individual expressions and unique language nuances would be removed during the transcription, analysis and writing phases to prevent identification. Only information pertinent to the findings was incorporated into the final thesis (Doody & Noonan 2016). Participants were informed that they could withdraw at any time during data collection, but the researcher would retain the data until withdrawal, as no identities were disclosed. Beneficence was upheld by asking participants questions that did not embarrass or discomfort them during the appreciative inquiry (AI) discussions. No harm was anticipated during data collection, and if any participant experienced mental distress, they would have been referred to a counselling professional, like PsyCAD, at their institution for support.

### Development of a model as a framework for promoting mental health support among radiation therapy students throughout their academic training programme

#### Step 1: Concept analysis

In this study, concept analysis was conducted in two steps, namely concept identification and definition and classification of concepts (Chinn & Kramer 2018; Walker & Avant 2011).

#### Step 1 – Phase one: Identification of the central concept

A thesis by Mokoena (2025) was conducted to: (1) explore RT students’ experiences working with patients in radiation oncology departments during their academic training and (2) explore the perceptions of RT lecturers and clinical tutors on how to best support RT students in these environments.

The study population included two purposefully selected groups. The first comprised 12 RT students from the host university (two second-year, six third-year and four fourth-year), aged 22 years old–31 years old, with 11 females and one male. Eight were placed in public and four in private radiation oncology departments. These students participated in World Café (WC) discussions.

The second group consisted of eight female RT lecturers and clinical tutors, aged 40 years old–60 years old, from all three universities offering undergraduate RT programmes, including private sector tutors. Their experience ranged from 11 to 38 years, and all held qualifications in both diagnostic radiography and RT. The roles among the participants varied: One was a part-time lecturer who also practised as a radiation therapist in a private oncology department. Two were full-time RT lecturers. One served as a clinical tutor responsible for both teaching clinical modules and supervising clinical skills training. The remaining four were employed at clinical training centres affiliated with the three universities.

### Data collection

To positively frame the research questions, an Appreciative Inquiry (AI) approach was adopted, using the original 4D phases (Discover, Dream, Design and Destiny) (Clossey, Mehnert & Silva 2011; Michael 2005). The AI process engaged RT students, lecturers and clinical tutors in these four phases to reflect on positive clinical experiences, envision meaningful mental health support, propose actionable measures and suggest practical strategies for enhancing student well-being and learning in radiation oncology.

Two primary methods of data collection were employed, utilising the Microsoft Teams platform. Focus group discussions (FGDs) were conducted with RT lecturers and clinical tutors in October 2022. The first FGD included five participants and lasted approximately 100 min. The second FGD, comprising three participants and lasted approximately 70 min.

In December 2022, WC discussions were held with RT students. Conducted online via Microsoft Teams, breakout rooms replaced physical tables, with three students per group. The WC format involved four structured discussion rounds of about 20 min each. A facilitator remained in each group to summarise earlier conversations for incoming participants, ensuring continuity. A final ‘harvest’ (Pagliarini, 2006) session brought together insights from all groups. This adapted WC format promoted rich student engagement and diverse perspectives, deepening the understanding of their clinical learning. Data saturation was achieved following these sessions.

### Data analysis

Data were analysed using Tesch’s method of qualitative data analysis, as outlined by Creswell (2013). This method facilitates the systematic organisation and interpretation of textual data, allowing for the extraction of meaningful patterns and insights. The analysis involved an inductive process of coding, whereby a list of codes was developed and iteratively applied to relevant segments of the data as they developed. This was followed by a process of consensual validation between the researcher and the independent coder, during which they discussed and refined the emerging themes and categories until agreement was reached. An analysis of the data identified four key themes, which are summarised in Table 1 alongside supporting verbatim quotes. The quotes contain the following abbreviations: WCP5 (World Café participant number) and FGD1P4 (focus group discussion number and participant number).

**TABLE 1:** Themes and verbatim quotes.

Themes	Verbatim quotes
1. Positive experiences within the clinical environment	‘I think for me. I actually feel happy and appreciative on a daily basis.’ (FGD1, P4) ‘Like the patient remembered me and all of that, and they even asked for me. So yeah, I think that is those are one of the positive experiences that we get to see when working with patients.’ (WC, P7)
2. Mental health support needed to strengthen the positive experiences	‘Yeah, I’m also backing them up in saying that there should be workshops that they can make for, for students who are finding it difficult to navigate in clinical practices.’ (WC, P9) ‘I try to have a debrief session with with each group … And so I spend a lot of time. In one-on-one situations, just talking through emotions and it’s not a set meeting; th’ey’ll just come in and want to tell me about something because they need to know how they’re supposed to deal with it.’ (FGD2, P1)
3. Mental health measures essential to achieve the students’ dreams	‘And I think sitting with the students and chatting with them is really, really it’s it’s a big thing.’ (FDG1, P4) ‘So, I think they really do need to reevaluate how they select a clinical tutor … Yeah, there’s actually a very good thing it’s, you know, selecting the clinical tutor because you obviously need a role model.’ (WC, P4)
4. Strategies for the implementation of mental health measures	‘… So I feel like a certain level of consistency is required. So if we are going to be attending, umm Wellness workshops, then it should be something that is consistently, not every day. Not all the time, but it should have some sort of follow up …’ (WC, P12) ‘I think with lecturers they can also move with the times … we are a technological generation, we are online, we learn new stuff. … We are working with technology … So we need to be learning what’s current and not what’s old, because you’ll find that with the syllabus we are learning all stuff that we no longer using and our technology is more advanced than what we are being taught in class. … So if we can find bridge the gap into also adjusting the syllabus.’ (WCP8)

*Source:* Mokoena, P.L., 2025, *A model to facilitate the mental health of radiation therapy students during their academic training*, University of Johannesburg, Johannesburg

FGDs, focus group discussions; WC, world café; P, participant.

**FIGURE 1:**
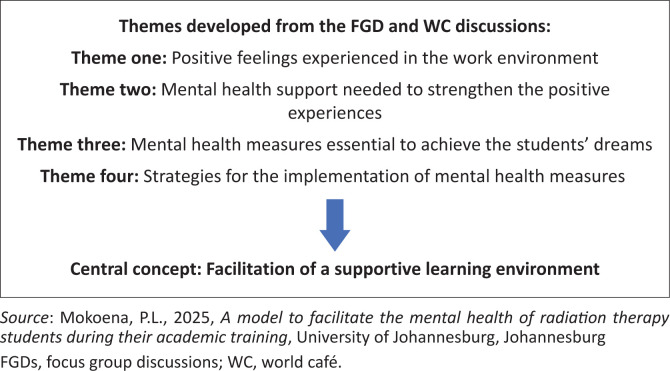
Themes of the radiation therapy lecturers, clinical tutors and radiation therapy students that emerged during the discussions as the basis for identifying the central concepts.

The findings revealed that participants found their involvement in patients’ care to be rewarding. Radiation therapy students felt valued within the radiation therapy team and shared that patients often remembered them long after treatment. Radiation therapy lecturers emphasised the importance of providing student support to ensure positive clinical experiences, particularly through regular debriefing sessions and access to professional counselling. Radiation therapy students expressed a strong desire for a nurturing learning environment that supports their professional growth. They highlighted the need for structured clinical training across departments, regular debriefing, expert counselling and workplace readiness sessions. Both students and lecturers also pointed to the importance of mentorship, role modelling, planned debriefings and training in advanced technology. Overall, it became clear that creating a supportive learning environment is essential for helping RT students reach their full potential and maintain good mental health throughout their academic training. Based on this, the central concept identified in the study was ‘facilitation of a supportive learning environment’ (see Figure 1).

#### Step 1 – Phase two: Definition and classification of concepts

In this phase, the central concept identified in phase one, ‘facilitation of a supportive learning environment’, was defined. To ensure a comprehensive understanding, definitions were sourced from various reputable online dictionaries, while theoretical perspectives were drawn from relevant literature, in accordance with the guidelines outlined by Chinn and Kramer (2018). These definitions were synthesised to construct a holistic conceptual overview. Through this process, both the essential and related attributes of the central concept were identified, culminating in a refined definition of ‘facilitation of a supportive learning environment’, as presented in this section.

Facilitation of a supportive learning environment is a process implemented by the RT lecturers and clinical tutors by mobilising resources to make things easier for RT students so that they are enabled and provided with help and encouragement when gaining knowledge or skills in the external conditions or surroundings where they live or work. This facilitation leads to a dynamic interactive process. The process of facilitation involves the RT lecturers and clinical tutors displaying behaviours that are helpful, encouraging, approachable, caring and accessible. The RT lecturers and clinical tutors use a range of skills and methods to offer RT students guidance and support during their academic training. They foster group cohesion and teamwork to bring the best out in RT students as they work to achieve the objectives of the academic training. This creates a safe and welcoming space for RT students to express their concerns, challenges or ideas. During this process, the RT students feel confident and empowered, and this leads to the promotion of RT students’ mental health, which is essential for coping and living a balanced life. This process aims to bridge the obstacles and provide RT students with a supportive environment that enables them to grow and develop personally and thrive while acquiring new and relatively enduring information and achieving their goals. Additionally, this process enables the RT lecturers and clinical tutors to develop respectful relationships with RT students. As a result, the facilitation process improves RT students’ mental health.

The central concepts were subsequently classified using the survey list developed by Dickoff et al. (1968). This survey list offered a systematic framework for the classification of concepts and involved the formulation of specific questions, as outlined in this section, which guided the classification procedure:

- *Who is the agent?* The RT lecturers and the clinical tutors are the agents and are responsible for facilitating a supportive learning environment.
- *Who is the recipient?* The RT students are the recipients who are provided with a supportive learning environment to meet their mental health needs.
- *What is the context?* The context is the environment in which the facilitation of the mental health of the RT students takes place. In this case, there are two contexts. The first one is the academic institution where RT students acquire new and relatively enduring information. The second context is the radiation oncology departments, where the RT students are placed for the clinical component of the qualification.
- *What are the dynamics?* The dynamics of this model reflect the expectations of RT students for their lecturers and clinical tutors to create a supportive learning environment. This includes clear communication, constructive feedback, debriefing sessions, and support for stress and mental health. Additionally, students seek to be heard and guided in their professional growth.
- *What is the process?* The process occurs when the RT lecturers and clinical tutors promote a supportive learning environment to strengthen the mental health of RT students during their academic training. This process involves the establishment of a positive and collaborative relationship between the RT lecturers, clinical tutors and RT students. Facilitation occurs when the RT lecturers and clinical tutors exhibit helpful, encouraging, approachable, caring and accessible behaviours.
- *What is the terminus?* The terminus is the outcome of the facilitation process. The outcome is the provision of a supportive learning environment for RT students during their academic training to strengthen their mental health.

#### Step 2: Describing relationships between the concepts

The central concepts identified in the first step were analysed for common characteristics, leading to the creation of relationship statements provided below:

- RT students have a range of requirements from RT lecturers and clinical tutors, and value a supportive learning environment where they are heard, feel connected and guided in their professional development.
- The RT lecturers and clinical tutors, through a dynamic integrative process, make it easy for the RT students to acquire or gain knowledge or skills in the external conditions, especially those in which the RT students live or work.
- The RT lecturers and clinical tutors’ facilitation process entails mobilising resources using a range of skills and methods to create a positive environment for the RT students and provide support and resources to achieve goals.
- The RT lecturers and clinical tutors enable and provide support to the RT students, who, as a result, gain confidence and competence and reach their personal and academic goals while also strengthening their mental health.

#### Step 3: Description of the model

This step involved the description of the model following Chinn and Kramer’s (2018) theory development. The six processes for the description of the model described by these authors were used to guide the model development process.

### Structure of the model

**FIGURE 2:**
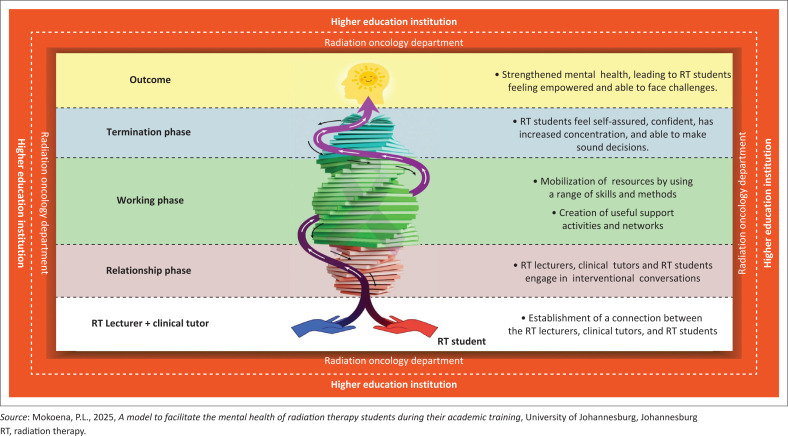
A model to support mental health of radiation therapy through the facilitation of a supportive learning environment during their training.

Figure 2 illustrates a visual representation of the model that promotes the mental health of RT students through facilitating a supportive learning environment during their academic training. The context is represented by a square-shaped border symbolising strength, stability and foundational growth, encompassing the two key environments where RT students are trained: the university (outer orange border) and the radiation oncology departments (inner brown border). The orange border symbolises encouragement, optimism and self-confidence, reflecting a supportive academic environment where students are motivated to engage actively with their lecturers. The brown border represents safety, dependability and security within the clinical training setting. A dotted line separates but also connects these two institutions, signifying their inseparable partnership in the holistic development of RT students. As the two colours merge near the dotted line into terracotta, this blend symbolises the comfort and warmth students experience through the integration of academic and clinical training, which is anticipated to ultimately provide a nurturing environment that supports their personal and professional growth.

At the base of the model are two open hands, symbolising the RT lecturers, clinical tutors and students reaching out in mutual openness and a willingness to engage in meaningful dialogue regarding the challenges encountered during academic training. The upward-facing arrow connecting the hands signifies a shared readiness to address these challenges and move forward collaboratively. The royal blue hand on the left represents the RT lecturers and clinical tutors, embodying qualities such as dependability, empathy, kindness and trustworthiness, characteristics essential for fostering supportive and trusting relationships. In contrast, the red hand on the right symbolises the frustration and emotional strain experienced by RT students during their academic journey. As the hands join, the connecting band transitions into purple, representing the merging of support and struggle, and signifying the development of mutual understanding. This purple band spirals around books, symbolising the deepening connection between students and facilitators, as well as their collective commitment to fostering a nurturing and supportive educational relationship.

The process of the model is represented by a spiral of books, symbolising the continuous facilitation of a supportive learning environment. The upward spiral reflects evolution, transformation and progressive growth, illustrating how RT students develop academically, clinically and personally throughout their training. Books were intentionally chosen to represent wisdom, knowledge and intellectual development, signifying the academic and clinical learning RT students acquire from both higher education institutions and radiation oncology departments. This learning fosters problem-solving, critical thinking and a deeper understanding of radiation therapy protocols. The open book at the top of the spiral signifies the free and ongoing sharing of knowledge, aimed at empowering students to succeed. The spiral’s dynamic structure also symbolises the interconnectedness of the model’s phases and the journey of self-discovery, as students navigate the challenges and growth inherent in their academic and clinical experiences. The inward and outward motion of the spiral represents the natural flow of development, reinforcing that learning is continuous, non-linear and deeply personal.

Within the spiral of books, white upward-pointing arrows are embedded along the winding path, symbolising the progressive journey of RT students through the various phases of the model for facilitating a supportive learning environment. These arrows, characterised by their twists and turns, reflect the non-linear nature of academic and personal growth. The colour white was deliberately chosen to represent hope, clarity, open-mindedness, enlightenment, excellence and new beginnings, qualities that the model aims to instil in students at each stage of their academic training. In contrast, black downward-pointing arrows depict periods of emotional difficulty, such as sadness, depression or demotivation, which may signal the need for RT lecturers and clinical tutors to pause and re-evaluate the support strategies being implemented. Encircling the spiral is a ribbon, representing the radiation oncology departments where students gain clinical experience. The ribbon symbolises cancer awareness and reinforces the broader context in which RT students are trained to deliver care, highlighting both the emotional and professional dimensions of their journey.

The structure of the model will be discussed using the framework proposed by Chinn and Kramer (2011), focusing on the purpose of the model, the underlying assumptions and a detailed description of the model’s process.

### Purpose of the model

The model is designed to serve as a framework of reference for RT lecturers and clinical tutors to promote RT students’ mental health through facilitating a supportive learning environment while working in radiation oncology departments during their academic training.

### Assumptions of the model

The model is based on the assumptions of the Theory for Health Promotion in Nursing, which sees the person holistically in interaction with the environment in an integrated manner (University of Johannesburg 2012). The assumptions are as follows:

- A person is an RT student who attends university lectures and gains practical experience in a Radiation Oncology Department, learning about treatment protocols and interacting with cancer patients. Furthermore, a person is a clinical tutor who assists the student in their education during clinical placements, while RT lecturers provide theoretical lectures at a university.
- Radiation therapy lecturers, clinical tutors and students are seen as holistic beings interacting with their internal and external environments. The internal environment includes physical, mental and spiritual health, while the external environment consists of the university setting and radiation oncology departments, where students engage with tutors, healthcare professionals and cancer patients. The social environment reflects interpersonal relationships shaped by social values and personal beliefs.
- Mental health is a dynamic interactive process in RT students’ environment that reflects their relative well-being status and contributes to or interferes with health promotion. It is crucial to emphasise that other personal circumstances might influence or have an impact on how RT students think, feel and act, as well as how they deal with stress. The interaction between RT students and their lecturers and clinical tutors provides students with resources to manage stressful situations, enabling them to function effectively and realise their abilities. In addition, RT students’ mental health is strengthened when their expectations of their RT lecturers and clinical tutors are met, and also when they are heard, feel connected with the RT lecturers and clinical tutors, and guided in their professional development.
- The promotion of the mental health of the RT students will take place in two contexts. The first one is the academic institution where the RT students acquire new and relatively enduring information and where this study was conducted. The second context is the radiation oncology departments where the RT students are placed for the clinical component of the qualification. These clinical departments are affiliated with the university where the study was conducted. The RT students need support regarding the transition between these two contexts.

### The process of the model

A supportive learning environment for RT students involves feeling appreciated, valued, included and empowered. This is fostered when RT lecturers and clinical tutors listen to students’ needs and guide their professional development. This section will explore three key phases of the facilitation process, which are interconnected and contain specific actions that contribute to a supportive learning environment: the relationship phase, the working phase and the termination phase.

The relationship phase represents the initial step in establishing a supportive learning environment, wherein RT lecturers, clinical tutors and students collaborate to develop meaningful connections. This phase centres on cultivating positive interpersonal relationships grounded in trust and mutual respect (Frisby, 2018). Such an environment is expected to support RT students in navigating academic and clinical challenges while simultaneously promoting a sense of being cared for and valued. The overarching aim of RT lecturers and clinical tutors during this phase is to empower students to achieve both academic and personal goals, while reinforcing each student’s sense of worth and belonging. The facilitation phase commences with welcoming and affirming interactions from RT lecturers and clinical tutors, using encouraging expressions such as ‘We are excited to work with you’ and ‘We want you to succeed.’ These initial engagements are followed by clear communication of the meeting’s purpose and expectations (Girija, 2012), as well as invitations for students to share their experiences, challenges and needs. By actively listening and validating students’ perspectives, lecturers and tutors foster open, two-way communication. This phase involves identifying students’ individual needs and collaboratively exploring strategies to support their academic journeys and enhance their mental well-being. Establishing rapport and trust in these interactions contributes to sustained, supportive relationships in which students feel respected, valued and motivated to succeed.

The working phase represents the second stage of the process, where RT lecturers and clinical tutors assume leadership roles. They mobilise resources and address challenges to provide guidance and support to RT students throughout their academic training. The objective is to establish valuable support activities and networks that create a safe and welcoming environment for students to express their concerns and ideas. By nurturing a positive atmosphere, RT lecturers and clinical tutors motivate students to achieve their goals and develop personally while acquiring enduring knowledge. During this phase, RT students communicate their needs to the lecturers and clinical tutors, initiating an interactive process to tackle their challenges. The RT lecturers and tutors exhibit empathy, listen attentively and respect students’ concerns while clearly articulating processes. They pinpoint problem areas, fostering a safe space for students to share their ideas and challenges, which enhances their mental health and confidence. Radiation therapy lecturers and clinical tutors engage in discussions with RT students about academic requirements and implement supportive strategies, leveraging various resources to effectively meet needs for open communication, mental health workshops and workplace readiness training.

The termination phase takes place when a supportive learning environment has been established, allowing RT students to feel both encouraged and accountable for their own learning and development. This environment thrives on collaboration among RT lecturers, clinical tutors and students, fostering a safe space for the open discussion of concerns and ideas. The relationship and working phases are ongoing and may require revisiting as students face new challenges. Once students achieve their objectives, lecturers and tutors gradually withdraw, empowering them to apply their skills and knowledge to future endeavours.

The outcome is the establishment of a supportive learning environment for RT students throughout their academic training, which is crucial for enhancing their mental health. An optimal mental health outcome for RT students is characterised by a sense of well-being in their thoughts, feelings and behaviours. They are equipped to manage the stresses associated with working in a radiation oncology department and to engage positively with patients, lecturers and their support network. Furthermore, these students engage in self-care practices that foster self-awareness and self-regulation. They recognise their strengths, positively influence patients’ lives, adapt to change, make challenging decisions and effectively manage their emotions. If needed, RT lecturers and clinical tutors provide ongoing support by adhering to key phases of the process. Ultimately, this process bolsters the mental health of RT students by cultivating resilience and adaptability as they navigate academic challenges.

#### Step 4: Evaluation of the model

The model underwent a thorough evaluation utilising the critical reflection framework established by Chinn and Kramer (2018). This comprehensive approach examines the practical utility of a theory in real-world applications. To facilitate this evaluation, the researcher prepared an informative PowerPoint presentation, which was conducted through Microsoft Teams. Seven evaluators participated in this session, together with two doctoral students and the researcher’s two supervisors. Each evaluator brought a unique perspective to the discussion. The presentation lasted approximately 30 min, during which the researcher elaborated on the model’s key features, rationale and potential implications. Following the presentation, the evaluators engaged in a constructive dialogue, providing their valuable feedback and insights. Much of this feedback was carefully considered and integrated into the final design of the model, enhancing its overall quality and applicability.

The evaluators, affiliated with various South African higher education institutions, brought a diverse range of expertise, including five individuals with doctoral degrees and two with post-doctoral credentials, collectively amounting to over 50 years of experience. Among them, four specialised in radiography, while three focused on nursing. Prior to the presentation, the researcher sent the evaluators a consent form along with a link to an evaluation questionnaire on Google Forms. This questionnaire consisted of two sections: the first gathered demographic information, while the second contained questions about the model design (described in this section), to guide the evaluators’ reflections. The following is a summary of the outcome of the evaluation. The concerns of the evaluators were attended to, resulting in the refined model, as presented and described earlier:

- *How clear is the model?:* The model’s clarity sparked a variety of opinions among the evaluators. Some found it clear, while others offered suggestions. Enhancements included a ribbon for radiation oncology, an open book on the spiral books and a sun positioned inside the brain. Descriptors and an explanation for the square context were added, along with downward-facing black arrows to illustrate the challenges faced by RT students.
- *How accessible is the model?:* One evaluator recommended that the model would be more accessible if it were published in booklet format. Another evaluator asserted that the accessibility of the model is largely contingent upon the researcher’s commitment to disseminating it effectively. Furthermore, one evaluator highlighted that the inclusion of clear explanations enhances accessibility, while another indicated that the practical aspects of radiography remain inadequately defined.
- *How simple is the model?:* The evaluators were optimistic that implementing the suggested amendments would simplify the model.
- *How general is the model?:* The evaluators noted that the model’s applicability is limited to RT education and training.
- *How important is the model?:* Collectively, the evaluators concurred that the proposed model possesses the capacity to support, empower and enrich the academic experiences of RT students during their training, thereby making a significant impact on the overall quality of their educational journey.

### Original contribution of the model

A model has been created to foster a supportive learning environment to promote the mental health of RT students during their academic journey. This framework addresses the challenges students face and aims to create a supportive learning environment that improves their well-being in both academic and personal aspects. Additionally, it fosters a teaching atmosphere that equips RT students with the necessary clinical skills while encouraging them to take initiative in their academic development.

### Limitations of the study

The model has not been implemented at this time. Information on the evaluation of the implementation of the model is thus not yet available.

### Recommendations

The model developed in this research serves as a valuable tool for RT lecturers and clinical tutors, providing guidance on creating a supportive learning environment that enhances the mental health of RT students.

## Conclusion

The primary objective of this study was to develop and evaluate a model to promote the mental health of RT students. This goal has been successfully achieved. In addition, the ethical considerations and trustworthiness measures that were adopted during this study to ensure the integrity of the process were provided. It is important to note that an in-depth discussion of the strategies for effectively implementing the model will be outlined in a future article.
